# Annotations

**Published:** 1888-12-22

**Authors:** 


### Annotations.

**A New Opening for Nurses.**

People who travel with their eyes open are often able to make suggestions which render travelling both safer and pleasanter for all who come after them. Journeys by sea are to some people very pleasant, and to others the dismalest and most miserable of penances. Especially is this the case when sea sickness adds its terrors to the way. Perhaps a long sea voyage yields its maximum of misery to women who cannot go half a mile from shore without feeling that both the outer world and their inner economy are bent upon having a game at "see-saw," "up and down." At such times all past pleasures, and all present comforts, and all future hopes are alike forgotten. The misery of the moment swallows up both past, present, and future. The concentrated essence of all imaginable wretchedness discharges itself upon the victim in a ruthless and hopeless deluge of indescribable helplessness and despair. At such a moment a kind woman's presence is desired above all things, and if, in addition to kindness, the woman possesses the skill of a trained nurse, there is imparted to the sufferer a sense of comfort and tender care which go far towards the cure of the sickness. Stewardesses every travelled lady knows. Who has had experience of a trained nurse as stewardess? It is passing strange that the thought of employing trained nurses in this capacity seems to have occurred to very few. An esteemed lady correspondent, who has just had the experience of a long voyage, is convinced that not only do the large passenger steamships which run between the various ports of the Old World as well as between the Old World and the New, offer excellent openings for experienced nurses who are good sailers, but also that the presence of such nurses as stewardesses on board would be invaluable to delicate lady passengers and little girls. The idea seems to us excellent, and we commend it for immediate application, both to the large companies who run passenger steamers and to nurses who may have a taste for adventure and who do not fear to seek it on the bosom of the deep.

**London Boy Ruffianism.**

The Secretary of State recently decided to remit a fine of £2 2s., with costs, which had been imposed by Mr. Paget upon Mr. Herbert Williamson, a Chelsea teacher, for inflicting corporal punishment upon a boy in a Board school. The subject of corporal punishment is one which interests not only every parent who has boys at school, and every school teacher who has charge of boys, but also the whole community of which boys form the coming generation. The question is, are boys to be disciplined or are they not? are they to be trained or are they not? The answer is one which does not admit of a moment's doubt. Boys must be trained, both for their own sakes, for their parents' sakes, and for the sake of the community. If there is to be a choice between over-discipline and under-discipline, it is better that they be over-disciplined. Whilst we are the last to advocate over-brain work, particularly in the case of Board school children, many of whom are perhaps under-fed, we must insist that discipline is a requisite which can on no condition be dispensed with. Any one who walks about much in the evening after dark in a London suburb will see for himself what large numbers of boys there are whose conduct is not that of mere rough village lads, but that of ill-trained, and apparently both mindless and conscienceless ruffians. The present writer, whose avocations only permit him to seek his constitutional in the evening, always takes care to provide himself with a good stout stick, and not seldom has he to threaten to use it, both in his own defence, and in that of others more helpless than himself. The growth of the metropolis seems to have acted injuriously upon the youths of the working class. Not only do they show no respect for their elders, and what the Catechism calls their "betters," but they seem to have no sense of shame, even when caught saying and doing shameful things. The discipline of schools should certainly not be relaxed. It is necessary, however, for teachers to bear two things in mind: First, that the flogging should be done with calmness, and not when the flogger is in a rage; and secondly, that there are certain parts of the human integument which may be thrashed with perfect safety. To these parts the teacher should always confine the stimulating attentions of his cane. In Scotland, school discipline is much more severe and much more efficient than in England. Very few Scotch boys in after-life will complain that they had too much of the Tawse in early youth.

**Funeral Wreaths.**

In the *Scotsman* of December 8th the following announcement appeared in the obituary: "At 5, South Gray Street, Newington, Edinburgh, on the 29th ult., Jane Smeal, aged 86, widow of John Wigham, Tertius. Wreaths affectionately declined." Mrs. Wigham was, we believe, a Quakeress, and had devoted her strength and much of her means to the service of the needy and the distressed. She was esteemed by the prosperous and loved by the poor. At her death it was to be expected that many would be anxious to show by some palpable sign their sincere esteem for the person of Mrs. Wigham, and their appreciation of her good works. Such a desire is perfectly natural, and the absence of it would be a sign of want of good sense and good feeling. But Mrs. Wigham's administrators, probably by Mrs. Wigham's desire, asked that no wreaths might be sent on the occasion of her death. Many people on reading this will feel a sense of gratitude and satisfaction. In many cases, however much wreaths may express the sympathy of the senders, they are terrible griefs to the recipients. Those who have had experience can call to mind how each knock at the door, each fresh letter of condolence, each additional wreath pierced the breast afresh, and made the agony of recent loss almost insupportable. In the case of well-known public persons, to whom the public owes recognition, the sending and receiving of wreaths or other tokens may be permitted—cannot, perhaps be avoided. But in ordinary instances, when a wife or a child or a parent has died, those who are left ought not to be constantly reminded of their sorrow, even by kind and sympathising acts. Death is a terrible mystery and experience to those who have to watch at the bedside of child or other deeply-loved relative or friend. At the moment, consolation, even of the most delicate kind, is, perhaps, impossible. The loud knock of the florist's messenger, the fresh card, the new wreath—these, as we have said, are to some people messages of the deepest anguish. The feeling of sympathy is natural. If it were not present, the heart that lacked it might consider itself cold and poor indeed. But it should prompt the friend, not to the indulgence of sympathetic feeling, but to the wish to spare those who mourn their dead any and every circumstance which can renew or aggravate their grief. With most people at such a time, the one desire is to be let alone. If any consolation is to be found, it is in the thought that the Parent of all spirits is the Parent of the living and the dead, and that the profoundest separations are mitigated and made endurable by His all-pervading and ever-living presence. The present custom of sending wreaths, which seems to be on the increase, is much to be deprecated on many grounds. It is not what a true simplicity would dictate; it adds an element of display which is foreign to the feeling of the moment; it sometimes imposes upon friends both anxiety and expense which they cannot afford; but, above all, it entirely fails of the end which friends have in view. However much some people may like to live in a crowd, no one possessed of the deeper feelings of humanity wishes to die in a crowd; still less do the living wish their beloved dead to be made the occasion of a public and expensive display.

